# Supplementary material for: A Thermokinetic Approach to Radiative Heat Transfer at the Nanoscale
Source: PLoS One. 2013 Mar 18;8(3):e58770. doi: 10.1371/journal.pone.0058770 (PMC3601111; doi:10.1371/journal.pone.0058770)
Supplement: Appendix S1 — Supporting Information. (PDF) [file pone.0058770.s001.pdf]

Supporting Information: Appendix S1

Agustín Pérez-Madrid, Luciano C. Lapas, J. Miguel Rubí

Lognormal distribution

This Appendix is devoted to the derivation of the expression of the lognormal distribution corresponding to Eq. (18) and also the stretched exponential function. The energy of the system results from the combined effect of a large number of independent inputs e_α, $e_1 + ... + e_n$. Let us assume that the effect on the energy of the input e_ν is proportional to e_ν and to the cumulative effect E_ν of the $\nu - 1$ previous inputs, $E_{\nu+1} = E_\nu + e_\nu E_\nu$. Whence

$$e_1 + ... + e_n = \sum_1^n \frac{E_{\nu+1} - E_\nu}{E_\nu} \simeq \frac{1}{k}\int_{E_1}^{E} \frac{d\epsilon}{\epsilon} = \frac{1}{k}\log\frac{E}{E_1} \tag{1}$$

For large n, the distribution of the sum is given by a Gaussian, according to the central limit theorem

$$\rho(\omega) \sim \exp\left[-\left(\frac{1}{k}\log\frac{\omega}{\omega_1}\right)^2 / 2\sigma^2\right] \tag{2}$$

where $E = \hbar\omega$. Equivalently

$$\rho(\omega)\, d\omega = \frac{k\omega_1}{\sqrt{2\pi}\sigma\omega}\exp\left[-\left(\frac{1}{k}\log\frac{\omega}{\omega_1}\right)^2 / 2\sigma^2\right] d\omega. \tag{3}$$

This distribution depends on two empirical parameters σ and ω_1.
